# *In vitro* optimization of crushed drug-sensitive antituberculosis medication when administered via a nasogastric tube

**DOI:** 10.1128/spectrum.02876-23

**Published:** 2023-11-22

**Authors:** Cassius M. Phogole, Jocelyn de Jong, Usha Lalla, Eric Decloedt, Tracy Kellermann

**Affiliations:** 1 Division of Clinical Pharmacology, Department of Medicine, Faculty of Medicine and Health Sciences, Stellenbosch University, Cape Town, South Africa; 2 Division of Pulmonology, Department of Medicine, Faculty of Medicine and Health Sciences, Stellenbosch University, Cape Town, South Africa; Foundation for Innovative New Diagnostics, Geneve, Switzerland

**Keywords:** HPLC, antituberculosis drugs, crushing, nasogastric tube, ascorbic acid, bioavailability

## Abstract

In low-resource settings, access to intravenous formulations of antituberculosis drugs is lacking or absent. Thus, critically ill patients with tuberculosis (TB) are often treated with readily available tablets which are crushed, mixed with water, and administered via a nasogastric tube (NGT). This has been associated with plasma concentrations below the recommended target, particularly of rifampicin (RIF) and isoniazid (INH), leading to clinical failure and development of drug resistance. We aimed to identify physiochemical factors contributing to lower plasma concentrations of antituberculosis drugs when administered in this manner using laboratory-based techniques, including the addition of ascorbic acid (AA) for optimum administration. Aqueous solubility of crushed medication using the inversion mixing method was evaluated against easily implementable mixing methods (sonication/vortexing) with or without AA. Stability of crushed medication in simulated-gastrointestinal fluids was evaluated with or without AA. Drug loss via NGT surface adsorption was quantitatively determined. RIF demonstrated poor aqueous solubility of 5.2%. Incorporation of AA improved the solubility to 29.7%, and with the addition of mixing techniques, solubility improved to 86.3%. Mixing methods with AA further improved the solubilities of both INH (80%) and pyrazinamide (PZA, 64%) to over 90%. AA also showed protective effects against unstable RIF in simulated-gastric fluid. Conventional flushing of NGT after drug administration with 10 mL of water resulted in drug losses of 1.5%, 0.13%, and 1.4% for RIF, INH, and PZA, respectively. An additional flushing step halved this amount. These interventions may improve the poor plasma concentrations of crushed antituberculosis drugs in critically ill patients.

**IMPORTANCE:**

The incidence of tuberculosis (TB) in intensive care units (ICUs) can be as high as 3% in high-burden settings, translating to more than 7,500 patients admitted to the ICU annually. In resource-limited settings, the lack or absence of intravenous formulations of drug-sensitive antituberculosis medications necessitates healthcare practitioners to crush, dissolve, and administer the drugs to critically ill patients via a nasogastric tube (NGT). This off-label practice has been linked to plasma concentrations below the recommended target concentrations, particularly of rifampicin and isoniazid, leading to clinical failure and the development of drug resistance. Optimizing the delivery of crushed drug-sensitive antituberculosis medication via the NGT to critically ill patients is of utmost importance.

## INTRODUCTION

South Africa (SA) is 1 of the 30 countries with the highest prevalence of tuberculosis (TB), accounting for 87% of the global TB burden (1). In 2022, SA had an estimated TB incidence rate of more than 500 cases per 100,000 individuals (2). It is not uncommon for patients with TB to be admitted to intensive care units (ICUs) (3 – 5). The frequency of bacteriologically confirmed TB in ICU is as high as 3% in high-burden settings and translates into more than 7,500 patients admitted to the ICU per year (6). Although acute respiratory failure remains the commonest reason for ICU admission, patients may present with alternative life-threatening conditions including TB meningitis, life-threatening haemoptysis, and adrenal insufficiency. Moreover, in high-burden settings, an incidental diagnosis of subclinical or asymptomatic TB is common in patients admitted to the ICU with conditions unrelated to TB including post-operative, trauma, or other medical conditions (6). The lack of access to intravenous (IV) formulations of drug-sensitive antituberculosis medication [rifampicin (RIF), isoniazid (INH), pyrazinamide (PZA), and ethambutol (EMB)], in low-resource settings, necessitates healthcare practitioners to crush, dissolve in water and administer the antituberculosis drugs to the patient via a nasogastric tube (NGT) (3, 7). Crushing of medication to administer via enteral feeding tubes, including NGT, is a widespread off-label practice in certain healthcare settings such as ICUs (8). This practice is necessitated by the lack of access to formulations for alternative routes of administration (8). Crushing, however, has been associated with plasma exposure of RIF and INH below the recommended target concentrations, which leads to poor clinical outcomes and the development of drug resistance (3, 9 – 11). The contributors to lower exposure are likely to be multi-fold including patient characteristics, concomitant diseases, pathophysiological changes unique to the critically ill patient, interactions with other co-administered drugs, and the delivery of enteral feeds (12 – 14). Separating concomitant interacting medicines and interrupting enteral feeds are potential methods to minimize interactions with crushed antituberculosis medicines (13). Although the solubility of crushed RIF in water is poor (15), water is still frequently used as an administration vehicle. Additionally, RIF has a relatively large molecular weight (>500 Da) and a structure featuring more than 5 H-bond donors and 10 H-bond acceptors meeting the criteria of Lipinski’s Rule of 5 which predicts sub-optimal oral activity (16). Therefore, improving the solubilization of poorly water-soluble crushed antituberculosis drugs may further improve their pharmacokinetics, including their bioavailability.

The solubility of poorly water-soluble drugs can be improved through various techniques, including modification of their chemical properties (salt formation, pH adjustment, and use of buffers), physical properties (particle size reduction, solid dispersion, and nanosuspension technology), and miscellaneous methods (solubilizer, hydrotrophy, surfactants, cosolvency, and novel excipients) (17, 18). The choice of a solubility-enhancing technique mainly depends on the properties of the drug, the site of absorption, and the characteristics of the desired dosage form (18).

We conducted a series of laboratory-based techniques focused on improving the solubility and stability of RIF, INH, and PZA with possible translational interventions for optimising administration at the bedside. The analytical analysis of EMB was excluded from this study, as it has adequate plasma exposure in most cases, including when the tablet formulation is crushed (9, 19). However, all drugs in this study were evaluated in the presence of EMB, since the treatment for drug-sensitive TB is a fixed-dose combination (FDC) of all these drugs during the intensive phase. The following properties of crushed RIF, INH, and PZA were evaluated: (i) aqueous solubility; (ii) stability when crushed and immersed in fasted-state simulated gastrointestinal fluids (FSSGIFs); and (iii) quantitative measurement of active pharmaceutical ingredients (APIs) lost by adsorption to the surface materials used for medication preparation and administration. The present study evaluated the solubility of crushed drug-sensitive TB medication in water using a cost-effective and simple hydrotropic method employing ascorbic acid (AA) with and without mechanical-resuspension methods of sonication and vortexing. AA is a water-soluble vitamin C with a well-established safety profile in humans (20, 21). AA was chosen as an intervention because the solubility of RIF is postulated to be low pH-dependent, and AA can lower the pH of water owing to its acidic properties. Additionally, AA is an antioxidant, and recent literature has shown the benefits of antioxidants in reducing gastric acid production,(22) which may enhance the stability of crushed drug-sensitive TB medication. Consequently, AA was also employed as an intervention for the stability assessment. Several studies have evaluated the antioxidant effect of AA in improving RIF stability, particularly in plasma samples where the drug is prone to oxidative degradation (23, 24). However, there is a paucity of data on the solubilizing and stabilizing effects of AA on crushed antituberculosis drugs in water and in an *in vitro* gastrointestinal environment. Moreover, data on AA interactions with other drug-sensitive TB drugs in the FDC *in vitro* gastrointestinal environment is limited.

## MATERIALS AND METHODS

### Reagents and chemicals

High purity reference standards of RIF (purity 98%), INH (purity 98%), and PZA (purity 98%) were obtained from Toronto Research Chemicals (Toronto, Canada). RIF (Rimactane capsule 150 mg, Sandoz, SA), INH (Winthrop-Isoniazid 300 mg, Sanofi-Aventis, SA), PZA (Macleods Pyrazinamide 500 mg, Macleods Pharmaceuticals, SA), and FDC (Rifafour e-275: RIF, INH, PZA, and EMB 150/75/400/275 mg, Sanofi-Aventis, SA), and Ryles tubes FG14 (Suprahealthcare manufacturing, Cape Town, SA) were donated by Tygerberg Hospital (Cape Town, SA). Ascorbic acid (AA), pancreas powder (lipase), pepsin, sodium chloride (NaCl) bioxtra, sodium hydroxide (NaOH) bioxtra, and potassium phosphate (KH_2_PO_4_) monobasic were purchased from Sigma-Aldrich (Missouri, USA). Ammonium formate (AF), acetic acid (AC), and formic acid (FA) were obtained from Fisher Chemical (Madrid, Spain). Ammonium acetate (AMAC) and hydrochloric acid (HCl) were purchased from Labchem (Johannesburg, SA). LC-MS grade acetonitrile (ACN) and methanol (MeOH) were obtained from Romil Pure Chemistry (Johannesburg, SA). A Milli-Q water purification system coupled with a Synergy UV system (Merck) was used to prepare ultrapure water. An ultrasonic bath (United Scientific) was used for the sonication of samples and mobile phases. Additionally, an Eins-Sci E-VM-A analog vortex mixer was employed to mix samples.

### Sample analysis

An i-Series Integrated High-Performance Liquid Chromatography (HPLC) System (Shimadzu) with LabSolutions Software was used to analyze RIF, INH, and PZA under the outlined chromatographic conditions listed in the supplementary data (Table 1).

**TABLE 1:** Chromatographic conditions

Analyte	RIF	INH	PZA
Mobile phase (A)(B)	Water: ACN (95:5, vol/vol) + 10 mM AF + 0.1% FA ACN: MeOH: Water (65:30:5, vol/vol/vol) + 10 mM AF + 0.1% FA	Water + 2 mM AA + 0.1% AC MeOH: Water (95:5, vol/vol) + 2 mM AA + 0.1% AC	Water + 0.1% FA ACN + 0.1% FA
Column	Venusil XBP C18 (2) (4.6 × 150 mm^2^, 5 µm)	Poroshell 120 EC-C18 (4.6 × 100 mm^2^, 2.7 µm)	Poroshell 120 EC-C18 (4.6 × 100 mm^2^, 2.7 µm)
Elution mode	Gradient: 55% B to 95% B over 6 min; 95% B to 55% B until 7 min, and re-equilibration until 11 min	Isocratic: 5% B	Isocratic: 60% B
Column temperature	Room temperature	Room temperature	Room temperature
Wavelength (nm)	334	264	265
Flow rate (mL/min)	0.500	0.550	0.350
Retention time	7.8	4.9	3.4
Run time (min)	11	6.5	5
Injection volume (µL)	10	20	5

### Statistical analysis

Statistical analysis was conducted using STATA version 17. A two-sample *t* test was employed for this purpose. GraphPad Prism version 8.0.2 was used for the graphical representation of the results.

### Determination of the aqueous solubility of the crushed drug-sensitive antituberculosis medication

A single INH and PZA tablet were independently crushed into a fine powder using a glass mortar and pestle. A RIF capsule was then carefully opened, and 2 mg of each powder was weighed out in duplicate. In the presence and absence of AA, the powder was reconstituted with distilled water to prepare stock solutions at a nominal concentration of 2 mg mL^−1^. However, prior to reconstitution with water, the mass of each sample was adjusted for purity (previously determined) to account for the presence of excipients in the formulation. The solutions were mixed using different methods as outlined in Table 2 (preparation of stock solutions from crushed RIF, INH, and PZA capsule/tablets for aqueous solubility evaluation), and the solubility of these drugs was also determined in organic solvents recommended in their Certificate of Analysis for reference purposes. The current study employed a manual inversion mixing method, owing to its cost-effectiveness, as an alternative to the commonly employed stirring mixing method using a mortar and pestle in clinical settings. The inversion mixing method, using only solvent water without AA, was used as a control to assess the impact of easily implementable interventions.

**TABLE 2:** Preparation of stock solutions from crushed RIF, INH, and PZA capsule/tablets for aqueous solubility evaluation^*a*^

Samples	1	2	3	4	5	6	7	8
Solvent	Water	Water with 1 mg mL^−1^ AA	Water with 20 mg mL^−1^ AA	Water	Water	Water	Water with 20 mg mL^−1^ AA	Organic
Mixing method	Invert manually	Invert manually	Invert manually	Sonication	Vortexing	Sonication and vortexing	Sonication and vortexing	Invert manually

^*a*^ Invert manually: 10 times; Sonication: set on normal mode at room temperature for 5 min; Vortexing: at a high speed for 30 s; Organic solvents used were MeOH and ACN, respectively, for RIF and PZA, as recommended in the certificate of analysis.

### Evaluation of the stability of crushed drug-sensitive antituberculosis medication in FSSGIFs

The stability of a drug product is clearly defined as its ability to maintain its physical, chemical, microbiological, and pharmacological properties within specified storage conditions (25, 26). A drug product is considered relatively unstable if it loses more than 10% of its potency during stability testing (26). Owing to the off-label practice of crushing drugs, most drugs have limited stability data when crushed. Thus, the aim of this experiment was to assess the effect of gastrointestinal pH on the stability of crushed drug-sensitive antituberculosis medication in the presence of gastrointestinal enzymes. This was accomplished by preparing FSSGIFs to mimic the fasted-state of TB patients during medication administration. In biowaiver studies, simulated gastrointestinal fluids (biorelevant media) have been widely used as representative models of the gastrointestinal environment to assess the stability, solubility, and permeability of APIs (27 – 29). The fluid composition of the gastrointestinal tract (GIT) varies along the tract and is affected by the patient’s prandial state (30). The simulated media of the prandial state can be prepared as FSSGF and FSSIF, which also represent the anatomical locations (31 – 33). In the postprandial state, the simulated medium should be prepared to mimic the fed state of the individuals (33).

Formulations of drugs are often designed in such a way that the APIs are delivered to the target site of the GIT where optimum absorption occurs and most of the oral drugs are well absorbed in the intestines (34). As a result, these APIs will need to be protected in the gastric environment from degradation by gastric acid before reaching the absorption site. Gastric transit time also plays a major role in this process as it determines the time the drug will spend in the stomach (34, 35). Although there is inter-individual variability in gastric transit time due to factors such as food intake, body posture, and age, the majority of people have gastric transit times of 0–2 and 2–6 h in fasted and fed states, respectively (35). Therefore, gastric transit time is another parameter that must be considered when assessing the impact of the gastric environment on APIs (35). The intestinal transit time is normally 3–4 h, while the bowel transit time is relatively longer, and can be up to 70 h (35). As a result, the simulated gastrointestinal fluids in the present study were prepared to imitate the fasted states of TB patients at the time of medication administration.

### Preparation of FSSGF at pH 1.2

FSSGF was prepared according to previously published protocols (15, 36, 37). Briefly, NaCl was dissolved in ultrapure water at a concentration of 34.2 mM. Afterward, HCl was added, and the contents were mixed with a magnetic stirrer. The pH of the solution was adjusted to 1.2 using a solution of NaOH. Pepsin enzyme at a concentration of 250 units mL^−1^ was added to the simulated solution before experimentation (38).

### Preparation of FSSIF at pH 6.8

FSSIF was prepared using an adjusted method as published by Marques (33), Pan et al. (36), and Santoveña-Estévez et al. (15). In summary, KH_2_PO_4_ was dissolved in ultrapure water at a concentration of 3.67 × 10^−6^ mol mL^−1^. The pH was adjusted to 6.8 with NaOH. Pancreas powder (lipase) at a concentration of 760 units mL^−1^ was added directly before experimentation (39, 40).

### Stability assessment of a whole versus a crushed drug-sensitive antituberculosis tablet (FDC) in FSSGIFs

#### Whole tablet

Beakers containing 25 mL of individual FSSGF and FSSIF were pre-incubated at 37°C for 15 min on a hot-plate magnetic stirrer before the addition of a whole Rifafour e-275 tablet to each beaker. These beakers were sealed with parafilm, and the speed of the magnetic stirrer was maintained at 250 rpm (41). In FSSGF and FSSIF, solutions were incubated at 37°C for 60 and 180 min, respectively, as recommended by the International Council for Harmonisation(42) guideline (2019). Samples were collected in triplicate from the FSSGF solution at 0, 30, and 60 min. The same protocol was followed for the FSSIF solution, but an additional sampling time point at 180 min was included.

#### Crushed tablet

The stability of the crushed drug-sensitive antituberculosis tablet in FSSGIFs was evaluated in the presence and absence of 4 and 20 mg mL^−1^ of AA (dosage: 80 and 400 mg). The tablet was crushed into a fine powder using a small beaker and pestle to avoid drug loss during the transfer from the mortar into a beaker. About 25 mL of pre-incubated FSSGIFs was added to the beakers containing the crushed tablet powder. The pestle used to crush the tablet was rinsed to reduce drug loss.

### Evaluation of the adsorption of drug-sensitive antituberculosis drugs to surface materials used in medication preparation and administration

The aim of this experiment component was to determine the potential amount of drug loss due to adsorption to the surface materials used during medication preparation and *in vitro* administration via NGT. The experiment was conducted as follows: a Rifafour e-275 tablet was crushed into a fine powder using a mortar and pestle and dissolved with 20 mL of distilled water. The suspension was thoroughly mixed for 60 s with the pestle. Using a 20-mL syringe, the suspension was flushed through an NGT mounted to a retort stand and clamp and positioned at a 30° angle, mimicking the general position of a supine patient (43, 44). The suspension mixture in the syringe was shaken adequately before delivery into the NGT to avoid the drugs adhering to the surface wall of the syringe. The flushed suspension was collected into a waste container. The mortar, syringe, and NGT were rinsed with 10 mL of water before the final rinsing step with 4 mL of organic solvent (MeOH: ACN, 50:50 vol/vol). The current study used organic solvents to ensure complete clearance of any potential APIs adhered to the surface materials. The mixtures of 4 mL of organic solvent were collected into separate containers for analysis on HPLC.

## RESULTS

### Aqueous solubility of the crushed drug-sensitive antituberculosis drugs

**Fig 1:**
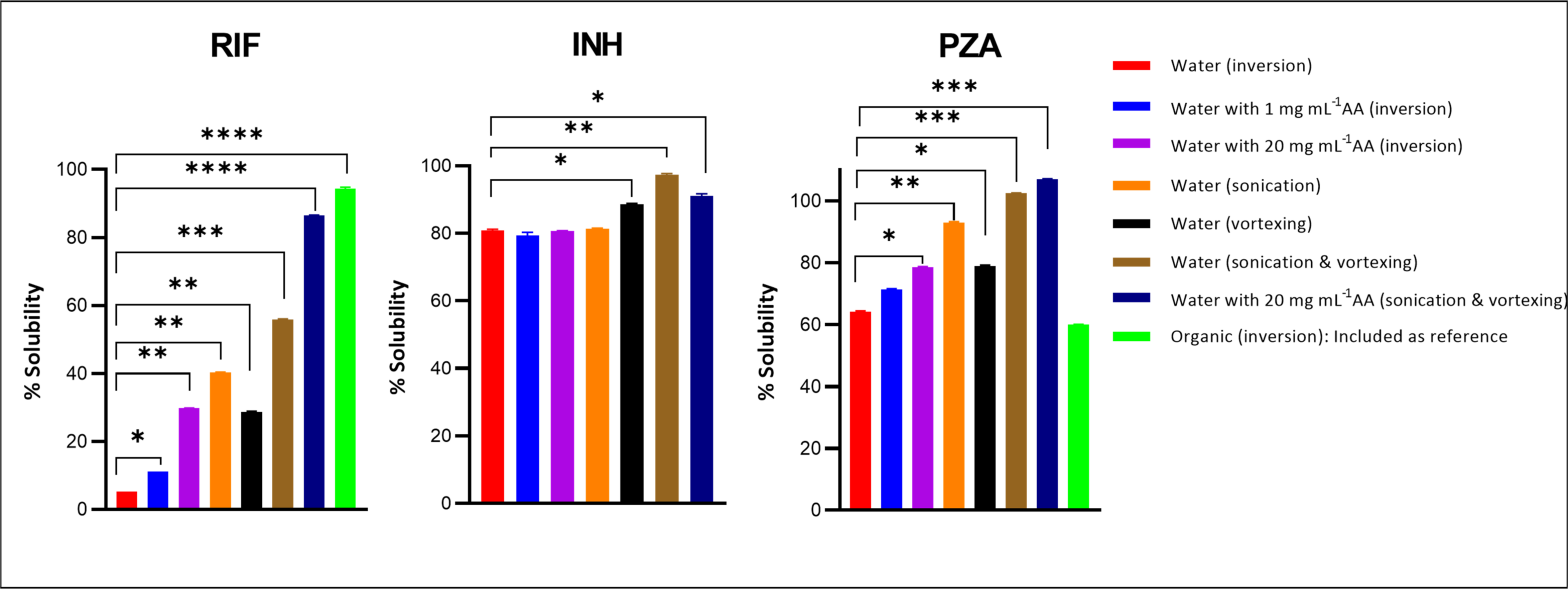
The aqueous solubility of crushed drug-sensitive antituberculosis medications in the presence and absence of AA (under varying mixing methods). The mixing methods included inversion for 10 times manually, sonication at room temperature in normal mode for 5 min, and vortexing at high speed for 30 s. The results are presented as the mean percentage ± standard deviation of six replicates, and the number of asterisk (*) indicates the statistical significance of the results (*P < 0.05; **P < 0.01, ***P < 0.001; ****P < 0.0001).

Figure 1 compares the aqueous solubility of crushed drug-sensitive antituberculosis drugs in the presence and absence of AA as a potential solubilizer, using varying mixing methods. Among the three study drugs, RIF had the lowest aqueous solubility of 5.2% when the inversion mixing method was used. Notably, the addition of AA at concentrations of 1 and 20 mg mL^−1^ significantly (*P* < 0.001) improved the aqueous solubility of RIF to 11.2% and 29.7%, respectively. The combined effect of sonication and vortexing achieved a 46.7% solubility of RIF, while the addition of AA at 20 mg mL^−1^ together with these mechanical interventions further improved the solubility of RIF to 86.3%. Moreover, the solubility of RIF in the organic solvent MeOH (included for reference purposes) using inversion mixing was 94.4%, indicating that RIF is poorly soluble in aqueous solutions. PZA in the present study demonstrated moderate aqueous solubility (64.2%, when mixed by the inversion mixing method). This was comparable to the solubility of PZA in the reference solvent, ACN, which was 60%. The combination of vortexing and sonication in the presence and absence of AA had a marked effect on PZA solubility, increasing it from 64.2% (using manual inversion) to 107% when used together with AA, and to 102.3% without AA. This implies the complete dissolution of the compound. This 107% solubility was due to the synergy between AA and mechanical resuspension methods, whereas 102.3% solubility was only due to mechanical resuspension methods alone. Using the inversion mixing method, INH was the only drug that demonstrated optimal aqueous solubility, surpassing the solubilities of both RIF and PZA, with 80.9% solubility observed in the absence of any interventions. However, when mechanical-resuspension methods were used in conjunction, INH solubility improved from 80.9% to 91.1% and 97.3%, respectively, in the presence or absence of AA. The solubility of INH in organic solvent was not investigated due to its high solubility in water.

### Stability assessment of whole versus crushed drug-sensitive antituberculosis tablets (FDC) in FSSGF

**Fig 2:**
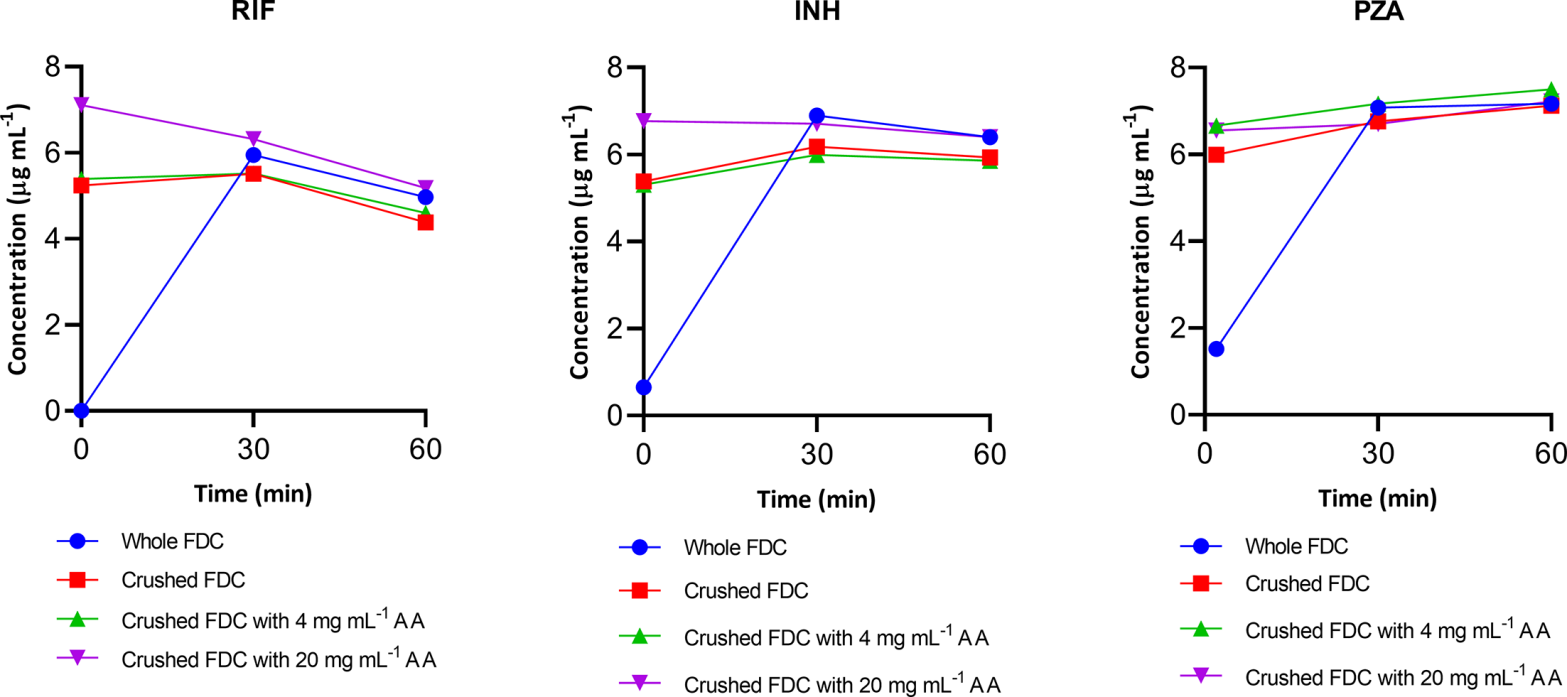
Stability comparison of whole versus crushed FDC (Rifafour e-275) tablets in fasted-state simulated gastric fluid (pH 1.2) at 37°C (*N* = 3)

During the FSSGF stability evaluation, both INH and PZA were unaffected by the presence or absence of AA, as they retained their concentration levels without any apparent degradation. However, RIF degraded in both whole and crushed FDC tablets, regardless of the presence or absence of AA (Fig. 2). Interestingly, as with the aqueous solubility test, AA at a higher concentration (20 mg mL^−1^) significantly (*P* < 0.05) improved the solubility of both crushed RIF and INH in FSSGF. Because of the enteric coating properties of Rifafour e-275 tablets, all drugs demonstrated *in vitro* lag times during the stability assessment of whole/solid tablets in FSSGIFs.

### Stability assessment of whole versus crushed drug-sensitive antituberculosis tablets (FDC) in FSSIF (pH 6.8) at 37°C

**Fig 3:**
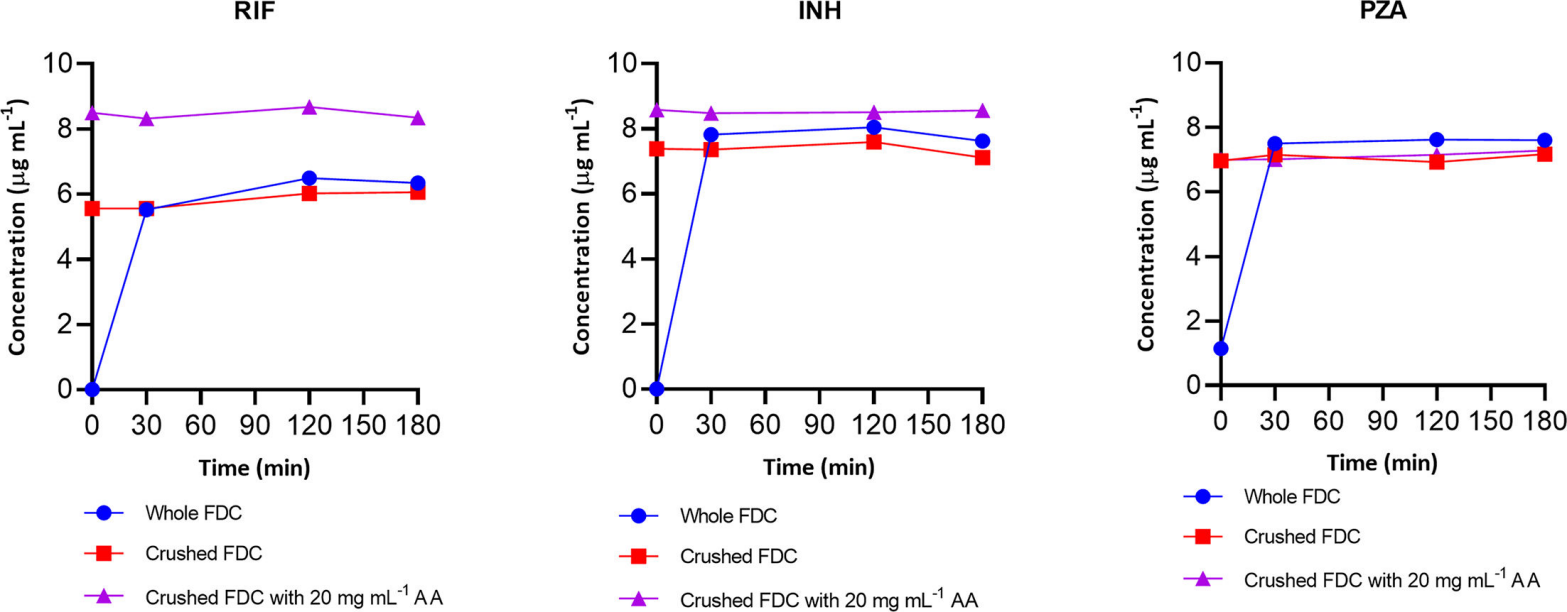
Stability comparison of whole versus crushed FDC (Rifafour e-275) tablets in fasted-state simulated intestinal fluid (pH 6.8) with and without AA (20 mg mL^−1^) at 37°C (*N* = 3)

Figure 3 shows the stability results of RIF, INH, and PZA in both whole and crushed FDC tablets with and without the addition of AA in FSSIF. All three drugs exhibited good stability in the presence or absence of AA (20 mg mL^−1^) in both whole and crushed tablet forms. Furthermore, AA significantly (*P* < 0.05) improved the solubility of both crushed RIF and INH in FSSIF, and a similar phenomenon was observed during solubility and stability (FSSGF) evaluations.

### Adsorption of drug-sensitive antituberculosis drugs to surface materials used in medication preparation and administration

A minimum recommended volume of water (10 mL) to rinse an NGT (mortar, pestle, and syringe) after medication administration was insufficient to elute all residue of crushed drug-sensitive antituberculosis drugs. The amount of API of the RIF, INH, and PZA lost by adsorption to the surface of the mortar and pestle after one rinse with 10 mL of water was 0.69, 0.039, and 2.5 mg, respectively, while the loss associated with the syringe was 0.25, 0.0021, and 0.085 mg, respectively, for RIF, INH, and PZA. For an NGT, the associated loss of the RIF, INH, and PZA was 1.4, 0.062, and 3.0 mg, respectively. As a result, the total mass of API of the RIF, INH, and PZA lost by adsorption after one rinse with 10 mL of water was 2.3, 0.10, and 5.6 mg, respectively, which equates to 1.5%, 0.13%, and 1.4% of the mass of API in the Rifafour e-275 tablet.

However, when two rinsing steps were employed in the current study, the amount of RIF, INH, and PZA lost by adsorption to the surface of the mortar and pestle after two rinsing steps (each step with 10 mL of water) was 0.0022, 0.022, and 0.97 mg, respectively. Drug adsorbed to the syringe was 0.098, 0.00, and 0.16 mg, respectively, for RIF, INH, and PZA. Moreover, an NGT was associated with the adsorption of 0.21, 0.018, and 0.39 mg, respectively, for RIF, INH, and PZA. The total amount of the RIF, INH, and PZA lost by adsorption to the surface materials after two rinsing steps (post *in vitro* administration) was 0.31, 0.040, and 1.5 mg, respectively. This translates to 0.21%, 0.053%, and 0.38%, respectively, of the total mass of API in the Rifafour e-275 tablet. As a result, the amount of APIs of crushed drug-sensitive antituberculosis drugs lost by adsorption to the surface materials used for medication preparation and *in vitro* administration through an NGT was significantly (*P* < 0.01) reduced after two rinses with 10 mL of water compared to a single rinse with 10 mL of water.

## DISCUSSION

The present study demonstrated that RIF is poorly water-soluble and is prone to degradation in simulated gastric fluid. The addition of AA at a relatively higher concentration (20 mg mL^−1^) significantly (*P* < 0.01) improved the solubility of RIF in both water and FSSGIFs compared to a lower concentration (1 mg mL^−1^). Furthermore, AA was also found to enhance the solubility of INH in FSSGIFs and PZA in water. The combined effects of sonication and vortexing further enhanced the solubility of drug-sensitive antituberculosis medication, particularly in the presence of AA. The solubility of RIF appears to be pH-dependent, with optimum solubility at low pH. Consequently, greater solubility of RIF was observed at a higher concentration of AA (20 mg mL^−1^, pH 2.5) than at a lower concentration of AA (1 mg mL^−1^, pH 3.1). This is consistent with findings by Chen (45) which showed pH-dependent RIF solubility, where maximum solubility was observed at pH 2. The poor aqueous solubility of RIF observed in this study is consistent with its biopharmaceutical classification system (BCS) as a class IV drug, characterized by low aqueous solubility and intestinal permeability (15, 46). This is further reflected in its partition coefficient (log *P*) of 2.7 (Table 3), which exceeds the ideal log *P* ≤ 1.8 of an orally administered drug (47).

**TABLE 3:** BCS class and log *P* of the first line antituberculosis drugs, adapted from Becker *et al*. (2009) (48) and Santoveña-Estévez et al. (15)^*a*^

Drug-sensitive anti-tuberculosis drugs	BCS (class)	Aqueous solubility	Intestinal permeability	Log *P* (partition coefficient)
INH	I	High	High	0.7
PZA	I	High	High	0.6
EMB	lll	High	Low	0.3
RIF	IV	Low	Low	2.7

^*a*^ Log *P* = [API in organic solvent]/[API in aqueous solvent], where [ ] represents the concentration. A negative Log *P* value indicates that the drug is hydrophilic, whereas a positive Log *P* value indicates that the drug is lipophilic (44, 46).

The aggregation of RIF capsule powder in water during the solubility assessment limited its solubility. Barbassa et al. (49) reported a similar observation, noting that the aqueous solubility limit of RIF is 1.3 mg mL^−1^ and at any concentration higher than this, the drug aggregates in an aqueous solution. In this study, test samples were prepared at a nominal concentration of 2 mg mL^−1^, which exceeded the established limit reported by Barbassa et al. (49). This aqueous solubility limit may explain the poor bioavailability of RIF observed in a pharmacokinetic study conducted by Koegelenberg et al. (3) at Tygerberg Academic Hospital in SA. In the study, ICU patients were administered crushed RIF (FDC) with water via an NGT at a nominal concentration of ≥15 mg mL^−1^. As a result, the solubility of RIF in the suspensions administered to patients was poor, despite patients being dosed according to their ideal body weights. In the clinical setting of an ICU, overcoming the aqueous solubility limit of RIF from crushed tablets is a challenge. The minimum RIF dose for adult patients is 300 mg daily, and to reach a RIF concentration of ≤1.3 mg mL^−1^, more than 230 mL of water is required to dissolve a crushed tablet, making it unfeasible for administration through an NGT. In contrast, the good aqueous solubility of INH observed in this study is consistent with its BCS as a class I drug, with the lowest log *P*, followed by PZA, EMB, and RIF, respectively (Table 3). This study further corroborates that PZA, another class I drug, also has optimal aqueous solubility.

The acid lability of RIF observed in this study is consistent with previous findings, which have reported degradation rates of up to 50% in stomach acid (15, 50 – 53). Moreover, co-administration with INH has been reported to reduce RIF stability in an acidic environment (51, 53). However, the effect of INH was not determined in this study, as FDC tablets were used. FDCs of drug-sensitive antituberculosis medications simplify treatment regimens and improve patient adherence. The stability of RIF supplemented with AA has been reported in previous studies (45, 53 – 55). Rajaram et al. (53) found that the stabilizing effect of AA on RIF in the presence of INH in an acidic medium is concentration-dependent; thus, higher concentrations of AA provide a greater stabilizing effect. The present study showed that a lower concentration of AA (4 mg mL^−1^) exhibited less protective effect towards RIF compared to a higher concentration (20 mg mL^−1^). The high stability of both INH and PZA observed in this study at physiological pH and temperature was previously reported by Santoveña-Estévez et al. (15). Moreover, the addition of AA did not appear to have a negative effect on the stability of either INH or PZA.

The present study demonstrated that there is a drug loss of drug-sensitive antituberculosis medication for administration through NGT after a single rinse with 10 mL of water compared to two rinses. The API of RIF adhered to the surface materials to a greater extent than INH, indicating that the relative insolubility of this non-polar drug in an aqueous environment result in it precipitating out of solution and potentially adsorbing to the surfaces of the NGT, syringe, and mortar and pestle. It was further observed with PZA, a drug with lower aqueous solubility compared to INH that its administration via NGT resulted in relatively high drug loss. This is supported by the findings of Zhu and Zhou (56), who reported similar phenomena for other non-polar compounds, such as amiodarone and phenytoin. These findings suggest that a single rinse of the NGT with 10 mL of water is insufficient to remove all APIs of the crushed drug-sensitive antituberculosis drugs. The present study showed that the overall percentage of drug loss by adsorption was not significant when compared to the total amount of API present in the dosage formulation. However, this could be far greater in a clinical setting due to the use of NGT for enteral feeding. The inner surface of the tube can become more adsorbent, resulting in greater adherence of medication to the surface if the tube is not adequately rinsed before administering medications. A survey study by Ekincioǧlu and Demirkan (57) showed that only 5–43% of health practitioners perform the rinsing procedure of the NGT before the administration of medication. To ensure sufficient delivery of the medication and achieve the therapeutic goal, it is recommended to rinse the NGT with an adequate amount of water before and after the administration of the medication (56, 58, 59).

In addition to its antioxidant and solubilizing effects on crushed drug-sensitive antituberculosis drugs, AA has been reported to potentiate the killing of *Mycobacterium tuberculosis* in both drug-susceptible and drug-resistant strains when used in conjunction with drug-sensitive antituberculosis therapy (60, 61). Furthermore, AA is a cheap and well-tolerated vitamin (61), in contrast to other additives (such as solubilizing agents) that have been linked to intestinal membrane complications (62). A recent pharmacokinetic study demonstrated the safety profile of AA at IV formulation dosages of up to 100 g with no iatrogenic outcomes (21).

Based on the findings of the current study, the addition of AA to crushed drug-sensitive antituberculosis drugs may be beneficial for TB patients administered crushed drugs, particularly within an ICU setting in the absence of alternative formulations. Because tablets are often crushed using a mortar and pestle and not an enclosed container, the use of a sonicator may be a more suitable option than a vortex mixer, which requires an enclosed container. Sonicators are devices that mix samples using ultrasonic waves and can be coupled with a noise-reduction enclosure to reduce high-frequency sound waves. They have been demonstrated to exhibit good efficiency in sample mixing, similar to a vortex mixer, and are cost-effective and user-friendly.

### Limitations

This study is not without limitations. To achieve cost savings, an inversion mixing method was employed instead of the commonly used stirring method with mortar and pestle, which does not fully replicate clinical practice. Furthermore, due to the use of a brand-new NGT instead of one that may have been used for enteral feedings, the drug delivery observed in this study may not accurately reflect the clinical setting, as the inner surface of the NGT could potentially be more adsorbent than what was observed in the study, affecting drug delivery to a larger degree.

### Conclusion

We found that sonication and vortexing, together with the addition of AA, can overcome poor solubility and stability associated with crushed TB drugs dissolved in water. Furthermore, when TB drugs are administered via an NGT, the crushing container and device, syringe, and NGT should also be washed with an additional 10 mL of water to minimize drug lost by adsorption to the surfaces. Human pharmacokinetic studies should be conducted to confirm our *in vitro* findings.

## SUPPLEMENTAL MATERIAL

Supplementary Methods spectrum.02876-23-s0001.docx Description of methods.

ASM does not own the copyrights to Supplemental Material that may be linked to, or accessed through, an article. The authors have granted ASM a non-exclusive, world-wide license to publish the Supplemental Material files. Please contact the corresponding author directly for reuse.
