# Supplementary material for: In vitro optimization of crushed drug-sensitive antituberculosis medication when administered via a nasogastric tube
Source: Microbiol Spectr. 2023 Nov 22;12(1):e02876-23. doi: 10.1128/spectrum.02876-23 (PMC10871698; doi:10.1128/spectrum.02876-23)
Supplement: Supplementary Methods — Description of methods. [file spectrum.02876-23-s0001.docx]

# Supplementary data

## Analytical method development and validation

Three individual reversed-phase HPLC methods for the quantification of RIF, INH, and PZA were developed and validated according to European Medicines Agency (EMA) (2011) and Food and Drug Administration (FDA) (2015) guidelines. Analytes were quantified using peak areas generated from the chromatographic conditions shown in Table 1.

### Preparation of calibration standards (STDs)

The reference standard was weighed out and adjusted for purity at a concentration of 2 mg mL¯^1^ (the reconstituting solvents for RIF, INH, and PZA were MeOH, water, and ACN, respectively). From this reference stock solution, STDs in duplicates at a concentration range of 1.56 - 100 µg mL¯^1^ were prepared. RIF STDs were prepared using MeOH containing AA at 20 mg mL¯^1^ to avoid oxidation, as previously reported (45, 52).

### Preparation of quality controls (QCs)

QCs were prepared at four different concentrations as follows: QC-High (80.0 µg mL¯^1^), QC-Medium (40.0 µg mL¯^1^), QC-Low (4.00 µg mL¯^1^), and lower limit of quantitation (1.56 µg mL¯^1^), by spiking reference stock solution (2 mg mL¯^1^) into the respective solvent for each analyte.

Assays validation

The developed analytical methods in the present study were shown to be specific, sensitive, robust, accurate, and precise. During the inter- and intra-day validation, the percentage accuracy and precision for all STDs and QCs were within acceptable limits for a valid calibration curve. Moreover, all analyte stock solutions demonstrated the absence of drug degradation during the stability tests conducted at room temperature for 24 hours, in a freezer (-80°C) for 4 weeks, in an autosampler for 24 hours, or on ice for 5 hours, which are the conditions that the test samples were anticipated to encounter during handling, processing, and analysis.
